# Estimation of Genetic Parameters for Heifer and Cow Fertility Traits Derived from On-Farm AI Service Records of South African Holstein Cattle

**DOI:** 10.3390/ani12162023

**Published:** 2022-08-10

**Authors:** Ramadimetje Delight Kgari, Carel Muller, Kennedy Dzama, Mahlako Linah Makgahlela

**Affiliations:** 1 Agricultural Research Council, Department of Animal Breeding and Genetics, Private Bag X2, Irene 0062, South Africa; 2 Department of Animal Sciences, University of Stellenbosch, Stellenbosch 7600, South Africa; 3 Department of Animal, Wildlife and Grassland Sciences, University of the Free State, Bloemfontein 9301, South Africa

**Keywords:** cow fitness, genetic improvement, heritability

## Abstract

**Simple Summary:**

Female fertility has gained significant attention and is incorporated in dairy cattle breeding objectives worldwide. Currently, age at first calving (AFC) and calving interval (CI) are the only traits used as indicators of fertility in the genetic evaluations of South African dairy cattle, but these traits are greatly affected by the breeder’s decisions and calving interval is available late in an animal’s life which delays selection decisions. This study investigated the possible use of additional fertility traits derived from artificial insemination records. The fertility traits examined in this study could be used in addition to age at first calving and calving interval to select for improved fertility in dairy cattle.

**Abstract:**

This study aimed to derive additional fertility traits from service data and estimate their genetic parameters for their possible inclusion in the South African Holstein cattle breeding programs. Service records (*n* = 64,464) were collected from 18 South African Holstein herds using on-farm automated milk recording systems. Using a multivariate model, the data were used to estimate heritabilities and correlations among several fertility traits. The pedigree data consisted of information on 18,592 animals born between 1981 and 2013. Heritability estimates observed were low to moderate (0.02 ± 0.00 to 0.24 ± 0.00), indicating that there is some genetic basis for the explored fertility traits to warrant selection. The genetic correlations observed between fertility traits were generally favorable, with some high correlations between age at first service (AFS) and services per conception for heifers (SPCh) (0.73 ± 0.00) and between days from calving to first service (CFS) and services per conception for cows (SPC) (0.90 ± 0.01). Positive genetic correlations indicate that improvement in one trait is coupled with a correlated genetic increase in another trait. The studied fertility traits could be used in addition to AFC and CI to serve as a basis for the selection of reproduction in dairy cattle to minimize selection bias.

## 1. Introduction

Dairy cattle farming aims to increase milk production at the lowest possible input cost. Profitability of dairy cattle not only depends on increased milk production but also on non-production characteristics such as improved fertility and health traits [1]. High-yielding cows that do not make it to the third lactation due to poor health or fertility will have lower lifetime daily yield than those reaching the fifth lactation with a lower average yield per day [2]. Poor reproductive performance consequently leads to high input costs associated with repeated inseminations, extra hormonal treatments for those cows failing to conceive, extended lactations leading to increased days open, and high inter-calving periods. However, due to milk being the produced commodity, dairy cattle selection programs historically put more emphasis on increased volumes with little or no emphasis on fertility traits [3]. In South Africa, estimated breeding values (EBVs) of Holstein cattle are routinely produced by national genetic evaluation centers for 5 milk production traits, 17 linear type traits, somatic cell score, and calving interval. A selection index known as the Breeding Value Index (BVI) was used in the past. The BVI was derived by a consensus approach, which is generally considered inappropriate as it lacks a scientific and economic basis and focuses on yield and type traits [4]. However, indexes that are scientific and economically balanced were developed and made available to the industry. These indexes include the traits of milk volume, fat yield, protein yield, somatic cell score, and calving interval.

Heritabilities for fertility traits are relatively low, indicating strong influence by environmental factors, which largely discouraged efforts for their genetic improvement through selection [5,6]. However, the pleiotropic effect of common alleles for fertility and production traits antagonistically resulted in the long-term deterioration in reproductive performance of high-yielding dairy cows [7,8]. Over the last two decades, many developed countries included female fertility in routine genetic evaluations and selection programs [6]. Several measurements such as age at first service (AFS), age at first calving (AFC), calving to first service (CFS), days open (DO), and calving interval (CI) are used as selection criteria for female fertility in genetic evaluations for many countries [9]. Inclusion of fertility traits in selection indices reversed earlier declining trends in fertility [10], with current improvements in calving intervals [11].

In South Africa, functional traits such as fertility are currently included in breeding programs. However, the improvement of fertility is based on AFC and CI, which are readily available from calving dates [12]. The selection for breeding animals in the Holstein herds is based on the estimated breeding values and the economic weighting of the traits. Although this is plausible, these fertility traits are more subject to biased management decisions such as determining when to start breeding for heifers or delaying breeding for high-yielding cows, which subsequently extends the DO and CI. Furthermore, CI is available late in an animal’s life, as it requires the first two calvings. Traits such as AFS and non-return rate are available earlier as selection criteria for heifer fertility, while CFS and DO [13,14] are better measurements commonly used for cow fertility. Although these traits are not routinely recorded under the South African National Milk Recording, this information is available on-farm through automated milk recording systems. These traits could be useful for the genetic improvement of female fertility in addition to calving traits. The objective of the current study, therefore, was to estimate heritabilities, as well as phenotypic and genetic correlations of female fertility traits derived from on-farm artificial insemination (AI) service records generated from automated milk recording systems in South African Holstein cattle.

## 2. Materials and Methods

The data for this study were collected from 18 South African Holstein herds. The data were collected from herds that use the Digital Information Management System South Africa (DIMSSA) program for performance recording. The records are not available on the National Milk Recording Scheme. They are available on-farm from the recording program and are used for herd management purposes. The herds used in this study are located across the country. South Africa’s climatic conditions generally range from the Mediterranean in the southwestern corner of South Africa to temperate in the interior plateau and subtropical in the northeast. These data stored in the farmer’s database included AI service records for 15,466 cows, where an outcome of each AI event was recorded, and the veterinarian-based pregnancy diagnosis using rectal palpation. Additional information included birth dates, service and calving dates of each animal, lactation number, and dam and sire identification numbers from which heifer and cow fertility traits were calculated. Table 1 shows the defined fertility traits that measured the ability of heifers to reach puberty early, the ability of cows to show heat early in the breeding period, and the probability of the success of insemination and confirmation of pregnancy. Non-interval traits were recorded as binary traits coded as 1 = no and 2 = yes. The extracted pedigree information consisted of 18,592 animals born between 1981 and 2013. Table S1 shows the kind of data used for analysis.

Data editing was carried out using the R-CRAN program (R Core Team, 2017). Two subsets of data were extracted from the original dataset for heifer (1st parity only) and cow (2nd parity and above). The datasets were edited to remove outliers for each trait. Removing outliers from the dataset included deleting records below 21 days or above 250 days for CFS and below 21 days or above 435 days for DO, as these biological anomalies could have been wrongly recorded. The biggest proportion of the dataset was lost during editing mainly due to missing pedigree records, as animals without parents were excluded. Furthermore, the animals that did not meet a biologically reasonable threshold for the traits were excluded from the final analysis. Two seasons of birth or calving were defined as summer (October–March) and winter (April–September). Contemporary groups were defined as the herd-year-season of birth or calving depending on the specific trait (e.g., herd-year-season of birth was defined only for the heifer traits AFS, AFC, and SPCh). After editing, the final data available for statistical analysis consisted of 10,019 and 24,909 records for heifers and cows, respectively. The pedigree data were edited to remove animals with unknown birth dates, with information remaining on 14,323 animals. The structure of the data before and after editing is shown in Table 2.

### Statistical Analysis

Descriptive statistics were computed using the core R functions and histograms were plotted using the Hist function in R [15]. The lme4 package [16] implemented in R-CRAN was used to test non-genetic factors associated with fertility traits. The analysis of variance (ANOVA) function in R was used to test the most significant effects associated with the traits. The effects tested were the herd-year season of birth or calving, age of insemination or calving, and the lactation number, which all affected fertility traits significantly (*p* < 0.05) and were included in the final models for estimation of genetic parameters.

The analysis for variance component estimation was first run using the AI REML of the BLUPF90 family of programs [17], which maximizes the likelihood with respect to parameters, yielding estimates of parameters corresponding to the maximum of that likelihood [18]. Multiple sets of bivariate analysis in AI REML yielded realistic results, while multivariate analysis gave an error owing to the over-parameterization of the model. The AI REML software does not allow simultaneous analysis of categorical and continuous traits. The results reported in this study were obtained from multivariate analyses using THRGIBBS1F90 and POSTGIBBSF90 of BLUPF90 [17]. The THRGIBBS1F90 uses Bayesian inference to estimate the unnormalized joint posterior distribution, where inferences are made on the marginal posterior distributions, in a Bayesian framework; the presence of nuisance parameters does not pose any formal, theoretical problems [19]. The Bayesian method implements a Markov chain Monte Carlo (MCMC) method and Gibbs sampling to estimate the marginal posterior densities of the different parameters [20]. The software allows simultaneous analysis of categorical and continuous traits considered in this study. Single chains of 300,000 cycles were run, with the first 50,000 cycles used as the burn-in period. This was followed by post-Gibbs analysis, using POSTGIBBSF90 to determine convergence by visual examination of plots of covariance components by iteration. Two sets of analysis were carried out: (1) estimation of 9heritabilities of cow fertility traits (DO, SPC, and CFS) and binary threshold traits (FS80d, PD100d, and PD200d), as well as their genetic correlations, and (2) estimation of heritabilities for heifer traits (AFS, AFC, and SPCh) and genetic correlations between heifer and cow fertility traits. The following animal models were fitted for heifer (SPCh, AFS, and AFC) and cow (SPC, CFS, DO, CFS80d, PD100d, and PD200d) traits:

(1) y=Xb+Za+e 

(2) y=Xb+Za+Wpe+e

where: **y** was the vector of observations; **b** was the vector of fixed effects which consists of herd-year-season of birth (for heifer traits), herd-year-season of calving (for cow traits), parity fitted only (for cow traits), age at insemination fitted only for (SPCh) and age at calving fitted (for cow traits) as a covariate; **a** was the vector of additive genetic effects; pe was the vector of random permanent environmental effects (fitted only for cow traits); **e** was the vector of residual effects; **X**, **Z**, and **W** were the corresponding incidence matrices. The expectations of E and variances were assumed as follows:

E(y)=Xb;E(a)=E(e)=0 and the var $\left(a\right)=A\sigma_{\;a}^{2}$ = G, var $\left(pe\right)=I\sigma_{\;pe}^{2}$ and var $\left(e\right)=I\sigma_{\;e}^{2}=$ R; therefore, var $\left(y\right)=ZAZ^{\prime }\sigma_{\;a}^{2}+WI\sigma_{\;pe}^{2}W^{\prime }+R,$ where A is the numerator relationship matrix.

The three-trait animal model was fitted as follows:

(3) $$\left[\begin{array}{c} y1\\ y2\\ y3 \end{array}\right]=\left[\begin{array}{ccc} X_{1} & 0 & 0\\ 0 & X_{2} & 0\\ 0 & 0 & X_{3} \end{array}\right]\;\left[\begin{array}{c} \beta_{1}\\ \beta_{2}\\ \beta_{3} \end{array}\right]+\;\left[\begin{array}{ccc} Z_{1} & 0 & 0\\ 0 & Z_{2} & 0\\ 0 & 0 & Z_{3} \end{array}\right]\;\left[\begin{array}{c} a_{1}\\ a_{2}\\ a_{3} \end{array}\right]\;\;+\;\left[\begin{array}{ccc} W_{1} & 0 & 0\\ 0 & W_{2} & 0\\ 0 & 0 & W_{3} \end{array}\right]\;\left[\begin{array}{c} pe_{1}\\ pe_{2}\\ pe_{3} \end{array}\right]+\;\left[\begin{array}{c} e_{1}\\ e_{2}\\ e_{3} \end{array}\right]$$

where $y_{i}\;$ was the vector of observations; I = 1, 2, and 3 representing fertility traits; $\beta_{i}$ was the vector of fixed effects for the ith trait, which consists of herd-year-season of birth (for heifer traits), herd-year-season of calving (for cow traits), parity fitted (for cow traits), age at insemination fitted for (SPCh) and age at calving fitted (for cow traits); $a_{i}$ was the vector of additive genetic effects for the  ith trait; $pe_{i}$ was the vector of random permanent environmental effects (fitted only for cow traits); $e_{i}$ was the vector of residual effects for the  ith trait; **X**, **Z**, and **WcdFGR** were the corresponding incidence matrices. 

Single chains of 200,000 cycles were launched in the THRGIBBS1F90, with the first 50,000 cycles used as the burn-in period during which the sampling process moves from the initial values of the parameters to those from the joint posterior distribution. This was followed by POSTGIBBS analysis to verify the burn-in period, determine the convergence by visual examination of plots of covariance components within each iteration and finally, the use of the posterior means to calculate heritabilities and genetic correlations. The variance components estimates from the single trait analysis were no different from the multi-trait analysis.

## 3. Results

### 3.1. Descriptive Statistics

Descriptive statistics of the data are presented in Table 3. The average AFS was 17 months while AFC was 27 months, which appears to be similar to international standards. The SPCh in heifers was lower (1.54) than in cows (2.18), indicating that younger heifers require fewer inseminations on average for conception than the older cows. This is somewhat expected as heifers have not yet started lactating to switch on genes underlying milk production.

### 3.2. Heritabilities, Genetic, and Phenotypic Correlations for the Derived Fertility Traits

Heritability estimates for all traits ranged from 0.02 ± 0.00 to 0.08 ± 0.01 in heifers and 0.04 ± 0.00 to 0.24 ± 0.00 in cows (Table 4). As expected, heritability estimates were generally low, except for the binary trait PD200d (0.24). The heritability estimates show that there is some genetic aspect to the traits to warrant improvement through selection. Positive genetic correlations of 0.90, 0.19, and 0.70 were observed between cow fertility traits SPC and CFS, SPC and DO, and CFS and DO, respectively. Positive genetic correlations indicate that improvement in one trait is coupled with a correlated genetic increase in another trait.

## 4. Discussion

The descriptive statistics in Table 3 show that, on average, it took 90 days for the cows to be ready for the first service after calving, which was higher than the 81 and 84 days reported for Holstein and Holstein–Friesian, respectively [21,22], although average CFS was lower than the 92 days for Holstein–Friesian reported by Tenghe et al. (2015) [23]. A high CFS interval leads to greater DO, which averaged 137 days in this study. This subsequently extends CI beyond the recommended 365 days, even with good heat detection and high pregnancy rates.

The heritability estimates ranged from low to moderate. Previously reported heritability estimates for AFS were 0.24 in Dutch Friesians [24], 0.12 in Canadian Holsteins [25], 0.22 in Ayrshire and Friesian cows [26], 0.13 in Canadian Holstein heifers [27], and 0.10 to 0.64 for Holstein cows in different herds in Germany [28]. These reported estimates were higher than the 0.02 observed in this study. Heritability of AFC (0.08) was comparable to 0.09 obtained for the Kenyan Ayrshire [29], although higher than 0.03 reported for Iranian Holstein cows using a single trait animal model [30] and lower than 0.13 obtained for multiple breeds of New Zealand using a bivariate approach on a sire model [31]. The differences could be due to the genetic variation among the populations, different statistical models used for analysis, varying reactions of the same breed to different environmental conditions, or differences in farm management strategies. The reliability of the data in terms of size and quality also influences the estimation of heritability. Age at first service is an economically important trait as it determines when an animal starts its reproductive life and influences AFC, which impacts generation interval and response to selection as it is closely related to rearing intensity. Both traits influence the lifetime productivity of cows. However, using AFS and AFC as reproductive measures has limitations as the decision to start breeding may be purely managerial. In addition, AFC excludes heifers failing to conceive, reducing available data for accurate genetic estimation of breeding values.

The SPC was defined for both heifers and cows, and the variance components differed considerably. The heritability estimate of SPC in heifers was slightly lower (0.02) than for the corresponding cow trait (0.04). These estimates were consistent with previous observations in the US Holsteins, Canadian Holsteins, Brown Swiss, and Chinese Holsteins [32,33,34]. The SPC can be a good indicator of fertility as it tells the breeder whether a cow is fertile or not, depending on the proficiency of the inseminator. Meanwhile, SPC is less dependent on management decisions in a viable dairy enterprise. Observed heritabilities for calving to first service and the number of days open were 0.06 and 0.05, respectively. Similar results were reported for Iranian Holsteins, Ireland Holstein–Friesian, and Chinese Holsteins [35,36] but were higher than the estimate of 0.03 reported for both Tunisian and Chinese Holsteins [37,38]. In contrast, higher heritabilities were reported for CFS (0.14) in Iranian Holsteins [30]. 

The current estimate for DO was similar to the values of 0.05 and 0.07 reported for Chinese [39] and Danish Holsteins, respectively [38]. However, higher heritabilities of 0.15 and 0.22, respectively, were reported for Ethiopian Holstein [40] and Holstein–Friesian [41]. Differences between the estimates of heritability observed in this study and estimates from other countries are most likely caused by different methods of estimation, management, and environmental factors that affect genetic and environmental variances. The interaction between the environment and genetics (G × E) plays a significant role in the expression of an animal’s full genetic merit in terms of performance [42]. 

The traits included in this study, excluding AFC, are not included in South African breeding programs due to a lack of information on the traits and because their estimated breeding values are not available on a nationwide basis. However, this study shows that the traits have the potential for inclusion in breeding programs. Several studies reported that DO should be preferred over CI in breeding programs for higher genetic progress because DO has higher heritability and genetic variance than CI [14]. This approach may be beneficial; an improvement in DO may lead to a correlated improvement in CI.

For success traits, heritability estimates ranged from low to moderate. The heritability estimate for the first service within 80 days post-partum was 0.07 and for the two binary traits indicating whether cows were confirmed pregnant within 100 or 200 days post-partum were 0.13 and 0.24, respectively. However, Potgieter et al. (2011) [43] observed lower heritability estimates ranging from 0.07 to 0.08 and 0.06 to 0.08 for both traits, respectively, using the same breed, although this study used multivariate analysis while Potgieter et al. (2011) [43] used a bivariate approach. The moderate heritability of the success traits indicates that a significant proportion of the variation in these traits is due to genetics. Thus, it may be efficient to improve the traits through selection. The success traits (PD100 and PD200) are not widely studied. However, their moderate heritabilities show the potential for their inclusion in fertility indexes. 

Results from this study were generally in agreement with previously reported estimates for fertility traits of relatively low heritability at 10%. The exception was cow success traits. Although we observed relatively low heritability estimates in this study, there is evidence of genetic basis in the analyzed fertility traits. Therefore, improvements in nutrition and reproductive management could be coupled with genetic selection for improved fertility in dairy herds. The heifer traits AFS and SPCh are available early in the animal’s life and could be used in addition to age at first calving as fertility indicators in genetic evaluations of the South African Holstein cattle population. High positive genetic relationships observed between SPCh, AFS, and AFC indicate that younger cows conceive from fewer inseminations. Lower AFS and AFC positively affect genetic progress as generation interval decreases and it allows early progeny testing of sampled bulls. Selection for decreased AFS and AFC may be an efficient strategy for dairy farmers to reduce costs [44,45]. Heifer traits could be useful in fertility indexes as they are available early in an animal’s life and are positively correlated with cow traits. The desirable improvement of heifer fertility performance could lead to a favorable reproductive performance for cows. As such, the studied traits have the potential to be included in national genetic evaluations and due to the traits being highly correlated genetically, positive genetic progress is expected from the use of such traits.

Genetic and phenotypic correlations were estimated amongst the defined AI service-based fertility traits (Table 3). The genetic correlations between heifer traits were high and favorable, with the highest correlation between AFS and AFC (0.91 ± 0.01) being close to one. This result indicates that AFS and AFC could be treated as the same trait due to one being heavily dependent on the other and that they probably have the same physiological basis. These results were consistent with correlations of 0.98 and 0.99, respectively, reported by Jagusiak and Zarnecki (2006) [44] and Brzáková et al. (2019) [12]. Jamrozik et al. (2005) [26] found a positive correlation between AFS and SPCh (0.28), although it was lower than the value found in the current study. In contrast, Guo et al. (2014) [37] reported a negative correlation between AFS and SPCh (−0.31). Variation in correlations in different countries may be due to different body conditions of heifers inseminated for the first time affecting their ability to conceive. 

Positive genetic correlations of 0.90, 0.19, and 0.70 were observed between cow fertility traits SPC and CFS, SPC and DO, CFS and DO, respectively. Although the correlation between SPC and DO was low (0.19), it was similar to the estimate of 0.21 reported by Zaabza et al. (2016) [46]. The estimated correlation between SPC and CFS indicates that selection for shorter calving to the first service will result in cows conceiving from fewer inseminations. The trait CFS could be used to represent cow fertility in selection indexes as it is closely related to both SPC and DO, indicating that fewer days between calving and first post-partum service results in fewer days open and the cow will conceive from fewer inseminations. The use of CFS instead of DO may minimize selection bias because using DO could exclude cows culled for not getting pregnant and CFS is available earlier than DO. Shortened CFS and DO mean shorter calving intervals, which will lead to increased productivity due to cows completing more lactation periods.

Genetic correlations between heifer and cow fertility traits were positive, but the scale was wide (0.05–0.95). SPCh for heifers had the lowest correlation with SPC for cows (0.05), indicating that the number of inseminations required for a heifer to conceive is not related to the number of inseminations required for conception in cows. These results were in agreement with Raheja et al. (1989) [25], who reported a low genetic correlation between services per conception in heifers and cows (0.01). However, age at first service and age at first calving showed high positive genetic correlations with cow SPC (0.84 and 0.95). Thus, early sexual maturity has a positive effect on the number of services required for conception later in the animal’s life. Genetic correlations of CFS and DO with all the heifer traits were moderate to high and positive (0.48–0.73). Previous literature reported moderate relationships between heifer and cow traits [47,48]. Abe et al. (2009) [46] pointed out that fertility traits for heifers and cows should be considered as different traits. Phenotypic correlations between heifer and cow fertility traits were generally close to zero (−0.13 to 0.03). Relationships among heifer traits were moderate to high and positive (0.28 to 0.89), with the exception of the estimate of −0.06 between SPCh and AFS. Phenotypic correlations among cow fertility traits were high and positive (0.42 to 0.71), with the exception of −0.15 for the phenotypic correlation between SPC and CFS. 

In this study, the genetic correlation of CFS with all the fertility traits was generally higher than the genetic correlations of DO with other fertility traits. The genetic relationship of heifer traits with cow traits indicates that heifer traits could be used in selection for improved fertility because these traits are available early in an animal’s life. Jansen (1987) [24] pointed out that records of virgin heifers and first parity cows could be useful in genetic evaluations to obtain a sufficiently accurate sire evaluation. Janson (1980)[49] found in Swedish Red and Whites that the non-return rate (56 days), as well as the number of inseminations per service period, was highly correlated in virgin heifers and first parity cows. Furthermore, heifer fertility traits were observed to be closely associated with production traits [7]. Wathes et al. (2014) [50] pointed out that aiming to rear replacement heifers to be bred at an early age (15 months) to calve at 24 months is optimum for economic performance as it reduces the non-productive period of cows while maintaining a seasonal calving pattern. This supports the current results that selection for early age at first insemination and age at first calving may be economically beneficial in dairy production. The estimated genetic correlations between fertility traits in heifers and cows were generally desirable, indicating that selection for improved fertility traits in heifers may improve reproductive performance later in the animal’s productive life. 

Estimates between cow interval and success traits were negative, ranging from −0.20 to −0.89 for genetic and −0.07 to −0.80 for phenotypic correlations, excluding a phenotypic correlation of 0.19 between SPC and FS80d. These correlations were generally favorable amongst the traits. The number of days open had the lowest genetic correlation with the number of cows being inseminated within 80 days post-partum (−0.20), although the relationship was favorable. Calving to the first service had the highest favorable genetic relationship with FS80d (−0.89), indicating that selection for a shorter interval from calving to the first service would result in a greater number of cows being inseminated within 80 days post-partum and more cows conceiving from fewer inseminations as the genetic correlation between FS80d and SPC is also favorable (−0.46).

The traits CFS, DO, and SPC had favorable relationships with PD100d and PD200d, suggesting that decreasing the number of inseminations and shortening CFS and DO will result in more cows confirmed pregnant within 100 or 200 days post-partum, which means a high percentage of cows will not exceed 200 days open. The genetic correlations of FS80d with PD100d and PD200d were positive and moderate (0.39 and 0.47), demonstrating that when more inseminations occur within 80 days post-partum, more cows will be confirmed pregnant within 100 days or 200 days post-partum. 

## 5. Conclusions

This study shows that fertility traits generally exhibit low heritabilities. Although heritabilities are low, they are non-zero, indicating that there is some genetic basis to warrant genetic improvement through selection. Heifer and cow fertility traits should be treated as separate but genetically correlated traits in multi-trait genetic evaluations. The high genetic correlations among the different fertility traits reveal the relationships amongst these traits, as predictions can be made on several fertility traits after performing selection on either one of the traits. Early availability of service data on fertility traits of heifers and the desirable genetic correlation with cow fertility traits presents an opportunity for these traits to be included in national genetic evaluations for South African Holstein cattle. The parameters estimated in the present study will facilitate the development of a genetic evaluation system for female fertility traits to improve the reproduction efficiency of South African Holsteins. Artificial insemination records provide an opportunity for the inclusion of additional fertility traits in genetic evaluations and potentially in the breeding objectives of the South African Holstein dairy cattle population. Therefore, farmers are encouraged to record such information and make it available for genetic evaluations in the quest to improve the reproductive performance of South African dairy herds.

## Figures and Tables

**Table 1:** Heifer and cow fertility traits are defined from on-farm service records.

	Trait	Trait Category	Description of the Trait
Heifer	Age at first service (AFS)	Interval	Age at which a heifer was first inseminated (expressed in months)
	Age at first calving (AFC)	Interval	Age at which a heifer gave birth to its first calf (expressed in months)
	Services per conception (SPCh)	Count	Number of services required for a heifer to conceive
Cow	Calving to first service (CFS)	Interval	Number of days from calving date to the date of the next first service
	Days Open (DO)	Interval	Number of days from calving date to conception date
	Services per conception (SPC)	Count	Number of services required for a cow to conceive
Cow	First service < 80 days (FS80d)	Success	Success trait—whether the cow was inseminated within 80 days post-partum
	Pregnant < 100 days (PD100d)	Success	Success trait—whether the cow was confirmed pregnant within 100 days post-partum
	Pregnant < 200 days (PD200d)	Success	Success trait—whether the cow was confirmed pregnant within 200 days postpartum

**Table 2:** Structure of the data.

Variable	Data before Editing	Data after Editing
No. of sires	8765	4668
No. of records (cows and heifers)	64,464	34,928
No. of records (heifers only)	34,678	10,019
No. of heifers with phenotypes	15,465	10,019
No. of records (cows only)	29,786	24,909
No. of cows with phenotypes	15,466	7072
Number of cows with records as heifers	10,537	6690

**Table 3:** Descriptive statistics of heifer and cow fertility traits.

	Trait	Mean	SD	Min	Max
	AFS (months)	16.8	3.5	10	30
	AFC (months)	26.7	3.9	20	48
Heifer	SPCh	1.54	0.98	1	8
	SPC	2.18	1.57	1	8
Cow	CFS (days)	90	37	21	250
	DO (days)	137	72	21	435
	FS80d	1.45	0.50	1	2
	PD100d	1.38	0.48	1	2
	PD200d	1.82	0.38	1	2

Age at first service (AFS); age at first calving (AFC); number of services per conception for heifers (SPCh); number of services per conception for cows (SPC); number of days from calving to first service (CFS); number of days open (DO); whether cows were inseminated for the first time within 80 days post-partum (FS80d); whether cows were confirmed pregnant within 100 days post-partum (PD100d) and whether cows were confirmed pregnant within 200 days post-partum (PD200d); Standard deviation(SD); Minimum (Min); Maximum(Max).

**Table 4:** Heritability estimates (on diagonal), genetic correlations (above diagonal) and phenotypic correlations (below diagonal) with standard errors of service-based heifer and cow fertility traits.

	AFS	AFC	SPCh	FS80d	PD100d	PD200d	SPC	CFS	DO
AFS	**0.02 ± 0.04**	0.91 ± 0.01	0.73 ± 0.00	0.27 ± 0.00	0.62 ± 0.01	0.04 ± 0.01	0.84 ± 0.00	0.36 ± 0.03	0.62 ± 0.00
AFC	0.89 ± 0.00	**0.08 ± 0.01**	0.69 ± 0.00	0.26 ± 0.00	0.42 ± 0.01	0.08 ± 0.01	0.95 ± 0.00	0.73 ± 0.01	0.63 ± 0.01
SPCh	−0.06 ± 0.01	0.28 ± 0.01	**0.02 ± 0.00**	−0.48 ± 0.01	0.30 ± 0.00	0.36 ± 0.00	0.05 ± 0.01	0.73 ± 0.00	0.48 ± 0.00
FS80d	0.01 ± 0.01	0.02 ± 0.01	0.01 ± 0.01	**0.07 ± 0.02**	0.39 ± 0.0.01	0.47 ± 0.0.01	−0.4.6 ±00	−0.89 ± 0.00	−0.20 ± 0.00
PD100d	−0.02 ± 0.01	−0.03 ± 0.01	−0.01 ± 0.01	0.37 ± 0.01	**0.13 ± 0.00**	−0.47 ± 0.00	−0.54 ± 0.01	−0.27 ± 0.00	−0.76 ± 0.00
PD200d	−0.01 ± 0.01	−0.02 ± 0.01	0.01 ± 0.01	0.12 ± 0.01	0.36 ± 0.01	**0.24± 0.00**	−0.57 ± 0.01	−0.63 ± 0.01	−0.85 ± 0.00
SPC	−0.13 ± 0.02	−0.12 ± 0.00	0.01 ± 0.01	0.19 ± 0.00	−0.48 ± 0.01	−0.58 ± 0.01	**0.04 ± 0.00**	0.90 ± 0..01	0.19 ± 0.01
CFS	0.12 ± 0.01	0.11 ± 0.01	0.03 ± 0.01	−0.71 ± 0.00	−0.42 ± 0.01	−0.25 ± 0.01	−0.15 ± 0.00	**0.06 ± 0.00**	0.70 ± 0.09
DO	−0.03 ± 0.02	−0.03 ± 0.01	0.01 ± 0.00	−0.29 ± 0.00	−0.07 ± 0.00	−0.80 ± 0.00	0.71 ± 0.00	0.42 ± 0.01	**0.05 ± 0.01**

Age at first service (AFS); age at first calving (AFC); number of services per conception for heifers (SPCh); number of services per conception for cows (SPC); number of days from calving to first service (CFS); number of days open (DO); whether cows were inseminated for the first time within 80 days post-partum (FS80d); whether cows were confirmed pregnant within 100 days post-partum (PD100d) and whether cows were confirmed pregnant within 200 days post-partum (PD200d).

## Data Availability

Data analyzed during this study are included in this Manuscript and its supplementary information files. In addition, more detailed data are available from the corresponding author on reasonable request.

## Supplementary Materials

The following supporting information can be downloaded at: https://www.mdpi.com/article/10.3390/ani12162023/s1. Table S1: performance data entailing derived fertility traits.
